# Epidemiology of Pediatric Meniscectomy: A Nationwide Study in Italy from 2001 to 2016

**DOI:** 10.3390/jcm11216259

**Published:** 2022-10-24

**Authors:** Umile Giuseppe Longo, Rocco Papalia, Alessandro Mazzola, Laura Ruzzini, Sergio De Salvatore, Ilaria Piergentili, Pier Francesco Costici, Vincenzo Denaro

**Affiliations:** 1 Research Unit of Orthopaedic and Trauma Surgery, Fondazione Policlinico Universitario Campus Bio-Medico, Via Alvaro del Portillo 200, 00128 Rome, Italy; 2 Research Unit of Orthopaedic and Trauma Surgery, Department of Medicine and Surgery, Università Campus Bio-Medico di Roma, Via Alvaro del Portillo 200, 00128 Rome, Italy; 3 Orthopedic Unit, Department of Surgery, Bambino Gesù Children’s Hospital, 00050 Rome, Italy

**Keywords:** pediatric, menisci, meniscectomy, sports medicine, arthroscopy

## Abstract

In the pediatric population, meniscectomy should be exceptional. This study aimed to estimate the incidence and trends of hospitalization of pediatric meniscectomy in Italy. Data were collected from the National Hospital Discharge Reports (SDO) reported at the Italian Ministry of Health. This study referred to the pediatric population (0–14 years of age) from 2001 to 2016. A total of 5188 pediatric meniscectomies were performed. The global incidence was 3.9 for every 100,000 Italian residents under 14 years of age. The most frequent age class was 10–14 years. The men/women ratio was 1.1. The average number of days of hospitalization was 1.8 ± 1.4. Men showed more days of hospitalization than women. The 5–9 age group presented the highest length of hospitalization (2.3 ± 1.5 days). The main primary codified diagnoses were as follows: derangement of the posterior horn of the medial meniscus, other derangement of the lateral meniscus, old bucket handle tear of the medial meniscus, and derangement of the lateral meniscus. Primary codified procedures were the excision of semilunar cartilage of the knee and knee arthroscopy. The burden of pediatric meniscectomy is relevant in Italy. The information required to develop global standards for managing pediatric meniscal lesions may be provided by conducting further epidemiological studies.

## 1. Introduction

Menisci are crucial structures of the knee, providing resistance to compression [1], stress absorption [2], and stabilization [2] and increasing the contact surface area within the joint [2,3]. Altering the meniscal structure may increase the incidence of associated injuries and cartilage degeneration within the knee, leading to early osteoarthritis [4,5,6,7]. Unfortunately, in the current literature, there is a lack of studies referring to the epidemiology of meniscal tears, treatment choices, patients’ features, and postoperative outcomes in the Italian population. Similarly, these data are also missing for the Italian pediatric population. Meniscal injury may occur up to 8.27 times per 1000 person-years in young, physically active individuals in the United States, ranging from 0.61 to 0.70 times per 1000 person-years in the overall population [8,9,10]. Meniscal disorders can range from severe tears in sports to discoid meniscal disorders in children. Older people frequently experience asymptomatic, degenerative tears [11,12]. Compared with the lateral meniscus, the medial meniscus is regularly two to three times more susceptible to injury [11].

Limited information has been published on the epidemiological trends and treatment approaches for meniscal tears in children and adolescents. However, meniscal lesions in the pediatric population have grown due to increasing sports activities among skeletally immature players [13,14,15]. Meniscal preservation and repair are crucial in younger athletes, where meniscal injury and resection can have long-term consequences [16]. Meniscal repair versus meniscectomy, in addition to the ideal time to treat young patients, is still being debated [17]. Arthroscopy is a successful therapeutic option for meniscal tears in children with a low failure rate, positive clinical results, and meniscal tissue preservation, according to recent research [14,17,18]. In contrast, the results of total or subtotal meniscectomy in pediatric patients are weak with early arthrosis [6,19].

However, the type of treatment depends on the lesion, age of the patient, and surgeon’s preference. Moreover, it is possible to observe different types of treatment among countries. For example, in the United States, arthroscopic partial meniscectomy after a meniscal tear is the most frequent orthopedic surgical procedure in adults [20]. On the contrary, few data have been reported about the epidemiology of these procedures in Europe.

Although several studies have analyzed the poor postoperative outcomes [14,16], limited knowledge is accessible regarding the epidemiological features of skeletally immature patients with a diagnosis of meniscal tear undergoing surgical meniscectomy. Unfortunately, we are unaware of any available database or registry on this population.

The present study aimed to determine the hospitalization trends and the incidence of pediatric meniscectomy in Italy from 2001 to 2016. The longitudinal analysis of national registers may help to obtain this information: they are crucial to adequately understand the burden of pediatric patients with potential orthopedic sequelae in the adult age, to guide the future operative indications and any associated health assistance plan.

## 2. Materials and Methods

To perform the analyses, the National Hospital Discharge records (SDO), a database provided by the Italian Ministry of Health, was used. The SDO database includes information from all Italian hospitals. This information is about patients’ features (age and sex), length of hospital stay, diagnoses, and procedures. Diagnoses are coded by the International Classification of Diseases, Ninth Revision, Clinical Modification (ICD-9-CM). Meniscectomy was defined by the following primary procedure codes: 80.6 and 80.26. The primary diagnose codes included were as follows: 717.2, 717.49, 717.0, 717.40, 717.3, 717.5, 717.41, 717.43, 836.1, 717.42, 836.0, 717.1, 717.89, and 717.9. ICD-9-CM Codes excluded from the database were reported in Table A1. Individual-level research data are available from 2001 to 2016. To analyze the incidence of meniscectomy in Italy, annual pediatric population data from the National Institute for Statistics (ISTAT) were used. The incidence rate was also stratified per year, age category, and sex. The study referred to the pediatric population (i.e., patients between 0 to 14 years).

### Statistics

IBM SPSS Statistics for Windows, version 26.0. (Armonk, NY, USA: IBM Corp.) and Microsoft Excel (2019) were used. Descriptive statistical analyses (frequencies and percentages for categorical variables and means and standard deviations for continuous variables) were used. The incidence rates of the procedures were calculated as the annual number of surgeries divided by the annual size of the pediatric population per 100,000 residents.

## 3. Results

### 3.1. Demographics

**Figure 1:**
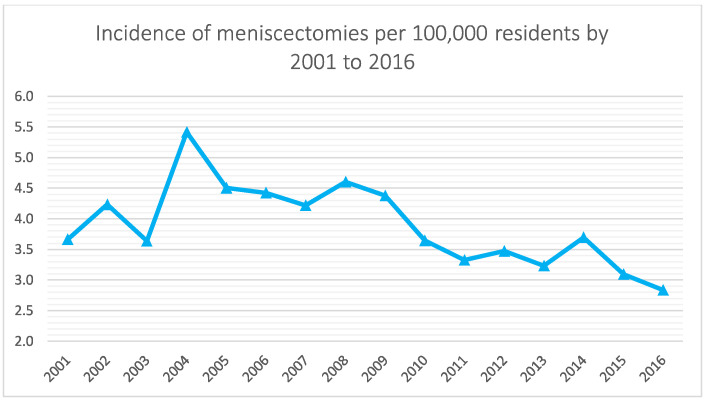
Incidence of pediatric (0–14 years of age) meniscectomy per 100,000 residents by 2001 to 2016 in Italy.

**Figure 2:**
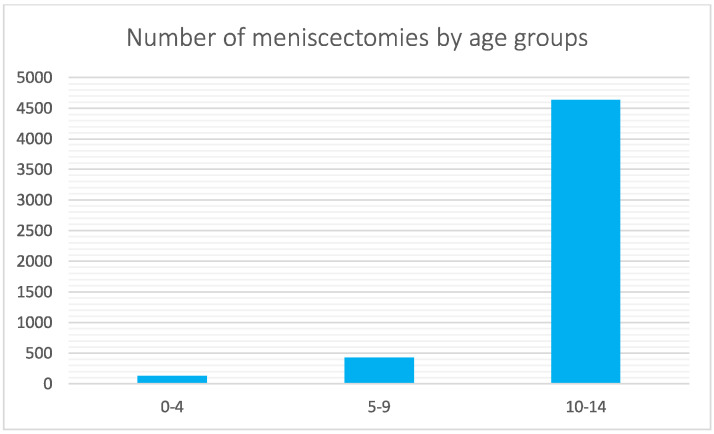
Number of pediatric meniscectomies performed in Italy from 2001 to 2016, stratified for class of age (0–4; 5–9; 10–14).

**Figure 3:**
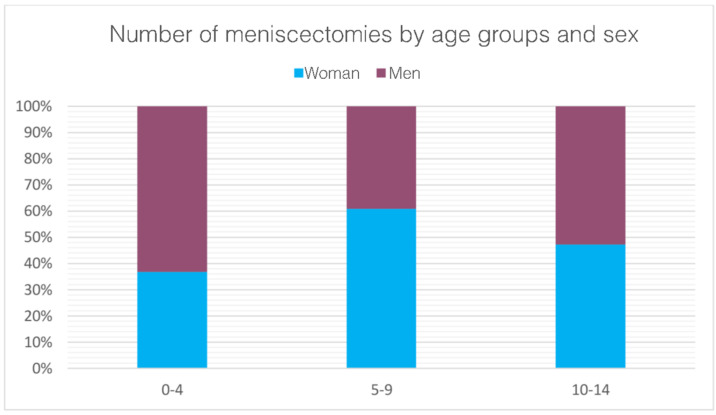
Pediatric meniscectomies in the study period stratified for class of age and sex.

**Figure 4:**
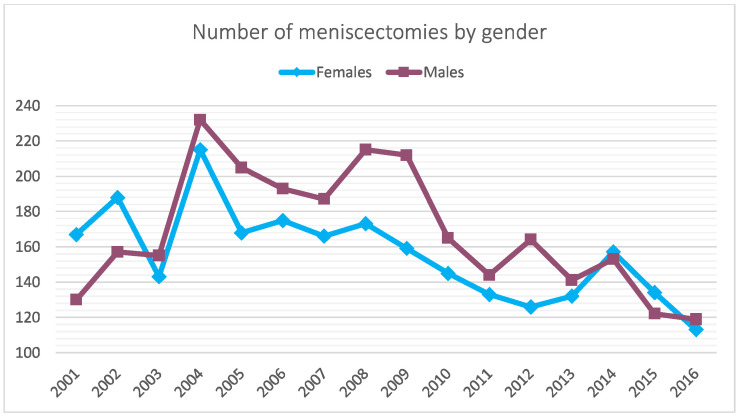
Sex distribution of patients requiring pediatric meniscectomy over the years of study period.

**Figure 5:**
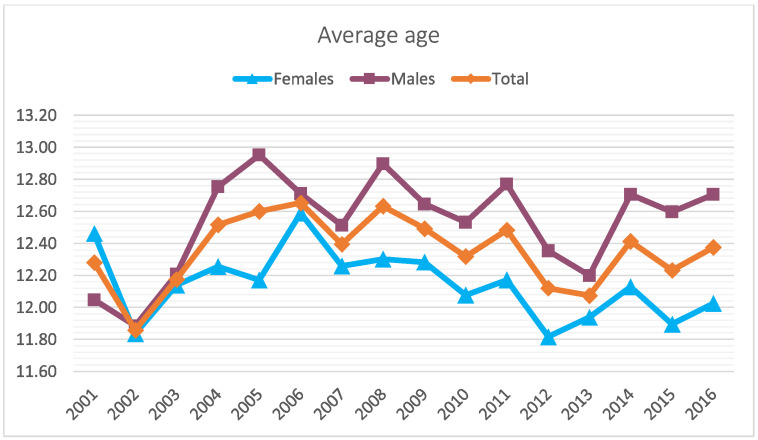
Average age of patients requiring pediatric meniscectomy over the years by sex.

Between 2001 and 2016, 5188 meniscectomies were performed in the Italian pediatric population. The global incidence rate was 3.9 procedures for every 100,000 Italian residents under 14 years of age. The incidence trend decreased from 3.7 in 2001 to 2.8 in 2016 per 100,000 residents, with a peak of 5.4 in 2004 (Figure 1). The most frequent age class was 10–14 years (89.3% of the patients) (Figure 2). During the 16-year period, the men/women ratio was 1.1. The percentage of men is slightly higher than women (51.9% and 48.1%, respectively). However, women were prevalent in the 5–9 age class (Figure 3). Moreover, in the two-year periods 2001–2002 and 2014–2015, women were prevalent (M/W ratio 0.8 in 2001 and 2002, 0.9 in 2014 and 2015) (Figure 4). From 2001 to 2016, the average age of the patients was 12.4 ± 2.7. Except in the year 2001, the men showed higher average age than women (Figure 5). Overall, the average age of women was 12.2 ± 2.6 and men 12.6 ± 2.8.

### 3.2. Length of Hospitalization

**Figure 6:**
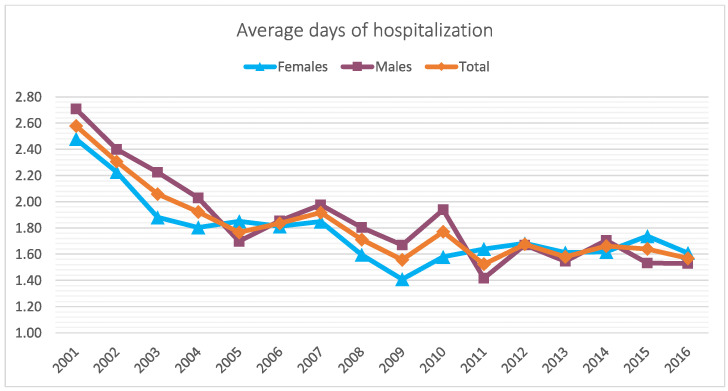
Differences in average days of hospitalization over the years by sex.

**Figure 7:**
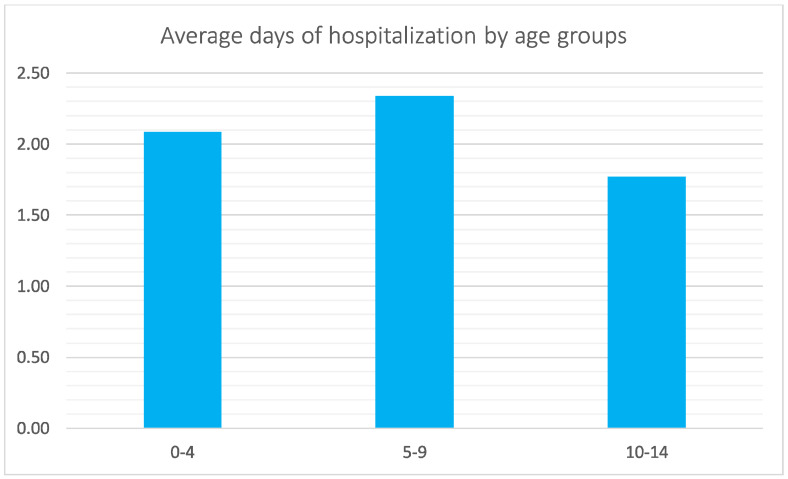
Average days of hospitalization by age groups in study period.

The average number of days of hospitalization was 1.8 ± 1.4. Between 2001 and 2016, the trend of the length of hospital stays decreased (2.6 ± 2.2 in 2001 and 1.6 ± 1 in 2016) (Figure 6). Overall, men showed more days of hospitalization than women (1.9 ± 1.5 for men and 1.8 ± 1.2 for women). The 5–9 age group presented more days of hospitalization with respect to the 0–4 and 10–14 age groups (2.1 ± 1.2, 2.3 ± 1.5, and 1.8 ± 1.3 days, respectively) (Figure 7).

### 3.3. Main Primary Diagnoses

**Figure 8:**
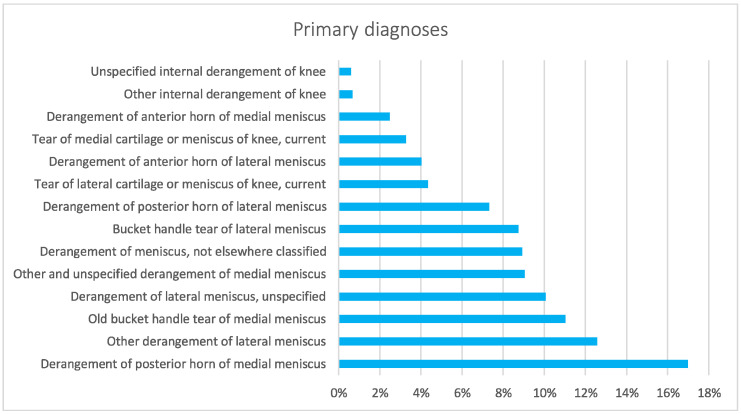
Main primary diagnoses (diagnoses are coded by the International Classification of Diseases, Ninth Revision, Clinical Modification (ICD-9-CM)) requiring pediatric meniscectomy from 2001 to 2016.

From 2001 to 2016, the main primary diagnoses were derangement of the posterior horn of medial meniscus (17%, ICD code: 717.2), other derangement of lateral meniscus (12.6%, ICD code: 717.49), old bucket handle tear of medial meniscus (11%, ICD code: 717.0), and derangement of lateral meniscus, unspecified (10.1%, ICD code: 717.40) (Figure 8).

### 3.4. Main Primary Procedures

**Figure 9:**
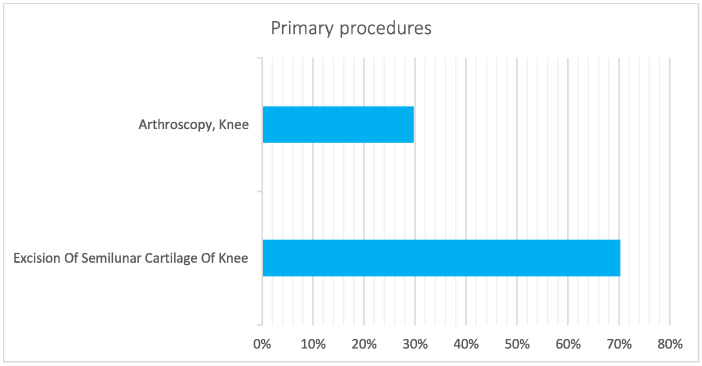
Main primary procedures for pediatric meniscectomy from 2001 to 2016.

During the 16-year study period, the primary procedures were excision of the semilunar cartilage of the knee (70.3%, ICD code: 80.6) and arthroscopy, knee (29.7%; ICD code: 8026) (Figure 9).

## 4. Discussion

Meniscal lesions represent a relevant group of diseases in the pediatric population [21]. Treatment strategies should be carefully evaluated for their long-term effects on the patient’s knee. When feasible, meniscal repair should always be preferred to both partial and total meniscectomy [16,18]. In the present study, pediatric meniscectomy showed a global incidence of 3.9 procedures for every 100,000 Italian residents under 14 years of age. It is known that only the peripheral 10–30% of the medial and 10–25% of the lateral meniscus receive adequate blood supply (the so-called “red–red” zone) [16]. The remaining two-thirds of the menisci nourish by diffusion (“red–white” and “white–white” zones) [16]. Compared with the adult cases, a more significant percentage of the meniscus is vascularized in children, making repair easier [18,22,23]. A longitudinal, vertical tear in the red–red or red–white vascular zone is the best tear shape for repair [16]. According to several authors, all lesions, independent of the type, should be repaired if they are reducible and the repair is stable [24,25].

The present study showed a decreasing incidence trend of pediatric meniscectomies in Italy over the study period. It may be attributed to an increased awareness of the poor long-term results of total and subtotal meniscectomy, encouraging meniscal preservation attempts [6,19]. Studies have shown the dangerous effects of the lack of the menisci in the biomechanics of the knee: total meniscectomy increases contact stress between the femoral condyle and tibial plateau up to 235% [19], where excision of even small bucket handle tears of the medial meniscus can increase contact stress by 65%.

When 75% of the posterior horn of the medial meniscus is resected, contact stresses are equivalent to total meniscectomy [19]. The majority of patients (89.3%) were between the ages of 10 and 14. Child begins playing sports more frequently at this age, which may contribute to the rise in meniscal injuries among patients in this group [26,27]. According to the literature, meniscal tears have been occurring more frequently in children [28,29], likely related to early sports specialty, year-round competition, and increased knowledge of and screening for these injuries. In the study period, the need for pediatric meniscectomy was almost the same for both men and women in Italy. Previous epidemiological studies have found significant sex differences in pediatric patients with meniscal tears regarding associated lesions: men were more likely to have lateral meniscal tears, posterior horn tears, peripheral tears, vertical tears, concomitant ACL tears, and an associated osteochondritis dissecans [21]. Female patients frequently showed medial meniscal tears, anterior horn tears, intrasubstance delamination, degenerative tears, discoid meniscus, and isolated meniscal tears [21]. From 2001 to 2016, a significant decrease in days of hospitalization after a pediatric meniscectomy was observed in Italy. For financial reasons, hospitals have probably performed a general reduction in the length of stay over the years, resulting in this difference. In terms of frequency, the results of the main primary diagnosis code analysis showed a higher involvement of the posterior horn of the medial meniscus in Italian pediatric patients (ICD code: 717.2). Medial meniscus tears have been included in the risk factors for treatment failure, together with complex and bucket-handle tears and skeletal immaturity [25,30,31,32]. Moreover, posterior horn tears have been reported to have inferior healing potential compared with lesions extending into the middle segments [33]. However, this result contrasts with other American studies that showed a higher involvement of the lateral meniscus in pediatric patients [21].

Our research has some limitations. Firstly, the ICD-9-CM, used in this study for all the reported diagnoses and procedures, is based on administrative data from different regions and hospitals. Therefore, identifying errors in diagnoses or coding due to the number of different hospitals involved is challenging. Second, several codes could be used for the same surgical procedure with the ICD-9-CM. For instance, the differences in coding between open and arthroscopic meniscectomy could cause our results to be underestimated.

## 5. Conclusions

In the pediatric population, meniscectomy should be exceptional to prevent long-term degenerative knee sequelae, namely the early development of knee osteoarthritis. Thus, management of meniscal tears has tended toward meniscal preservation. This study aimed to estimate the incidence and trends of hospitalization of pediatric meniscectomy in Italy based on official information sources such as hospitalization records. Overall, men showed more days of hospitalization than women. The 5–9 age group presented more days of hospitalization. The most frequent age class was 10–14 years. From 2001 to 2016, the main primary diagnoses were “derangement of the posterior horn of medial meniscus”, “old bucket handle tear of medial meniscus” and “derangement of lateral meniscus, unspecified”. This study confirmed that the socioeconomic burden of meniscal surgery heavily affects the pediatric population (0–14 years of age). These findings reinforce the need for strategies to improve access to more preserving meniscal treatment such as meniscal repair and healing.

## Data Availability

The datasets used and/or analyzed during the current study are available from the corresponding author on reasonable request. The access to the database is on request. All data were obtained by the Direzione Generale della Programmazione Sanitaria—Banca Dati SDO of the Italian Ministry of Health.

## Abbreviations

SDO—The National Hospital Discharge records; ISTAT—The National Institute for Statistics; ACL—Anterior Cruciate Ligament; ICD-9-CM—The International Classification of Diseases, Ninth Revision, Clinical Modification.

## Appendix A

**Table A1:** ICD-9-CM Codes excluded from the database.

Code	Frequency	Percent	Valid Percent	Cumulative Percent
1710	1	,2	,2	,2
2137	1	,2	,2	,3
2138	1	,2	,2	,5
2728	2	,3	,3	,8
47412	1	,2	,2	1,0
7062	1	,2	,2	1,1
7094	1	,2	,2	1,3
7103	1	,2	,2	1,4
71106	1	,2	,2	1,6
7142	1	,2	,2	1,8
71433	1	,2	,2	1,9
71516	1	,2	,2	2,1
71536	2	,3	,3	2,4
71666	1	,2	,2	2,6
71680	1	,2	,2	2,7
71686	2	,3	,3	3,0
71698	1	,2	,2	3,2
7176	11	1,8	1,8	5,0
7177	30	4,8	4,8	9,8
71782	4	,6	,6	10,4
71783	136	21,8	21,8	32,2
71784	2	,3	,3	32,5
71785	3	,5	,5	33,0
71800	1	,2	,2	33,2
71808	14	2,2	2,2	35,4
71810	1	,2	,2	35,6
71826	1	,2	,2	35,7
71846	5	,8	,8	36,5
71886	7	1,1	1,1	37,7
71898	1	,2	,2	37,8
71926	6	1,0	1,0	38,8
71940	2	,3	,3	39,1
71946	12	1,9	1,9	41,0
71948	22	3,5	3,5	44,6
71966	1	,2	,2	44,7
71996	3	,5	,5	45,2
72669	2	,3	,3	45,5
72700	10	1,6	1,6	47,1
7272	1	,2	,2	47,3
72740	2	,3	,3	47,6
72741	1	,2	,2	47,8
72749	1	,2	,2	47,9
72751	1	,2	,2	48,1
72761	1	,2	,2	48,2
72783	15	2,4	2,4	50,6
72789	13	2,1	2,1	52,7
7285	1	,2	,2	52,9
72889	3	,5	,5	53,4
72931	1	,2	,2	53,5
7321	1	,2	,2	53,7
7324	3	,5	,5	54,2
7326	1	,2	,2	54,3
7327	27	4,3	4,3	58,7
7328	3	,5	,5	59,1
7329	11	1,8	1,8	60,9
73343	1	,2	,2	61,1
73381	1	,2	,2	61,2
73392	9	1,4	1,4	62,7
73641	2	,3	,3	63,0
75559	1	,2	,2	63,1
75564	3	,5	,5	63,6
7569	4	,6	,6	64,3
7834	1	,2	,2	64,4
82121	1	,2	,2	64,6
8220	1	,2	,2	64,7
82300	8	1,3	1,3	66,0
82310	1	,2	,2	66,2
82322	1	,2	,2	66,3
8244	4	,6	,6	67,0
8362	22	3,5	3,5	70,5
8363	7	1,1	1,1	71,6
83650	1	,2	,2	71,8
8440	2	,3	,3	72,1
8441	3	,5	,5	72,6
8442	140	22,4	22,4	95,0
8448	3	,5	,5	95,5
8449	19	3,0	3,0	98,6
8676	1	,2	,2	98,7
9054	2	,3	,3	99,0
9597	1	,2	,2	99,2
9953	1	,2	,2	99,4
V4289	1	,2	,2	99,5
V540	2	,3	,3	99,8
V548	1	,2	,2	100,0
Total	624	100,0	100,0	
